# (Cyclo­pentane-1,1-di­yl)dimethanol

**DOI:** 10.1107/S1600536809001330

**Published:** 2009-02-06

**Authors:** Richard Betz, Peter Klüfers, Peter Mayer

**Affiliations:** a Ludwig-Maximilians Universität, Department Chemie und Biochemie, Butenandtstrasse 5–13 (Haus D), 81377 München, Germany

## Abstract

In the title compound, C_7_H_14_O_2_, co-operative eight-membered homodromic rings of O—H⋯O hydrogen bonds connect the mol­ecules into strands along [100]. According to graph-set analysis, the descriptor of these cycles is *R*_4_^4^(8). The cyclo­pentane-ring adopts an envelope conformation (^*C*4^ *E*).

## Related literature

The compound was synthesized according to a published procedure (Domin *et al.*, 2005 ▶). For the influence of chelation to (semi-)metals on the geometry of bifunctional alcohols, see: Klüfers & Vogler (2007 ▶). For the structure of a related compound, see Wender *et al.* (1999 ▶). For details on graph-set analysis of hydrogen bonds, see Etter *et al.* (1990 ▶); Bernstein *et al.* (1995 ▶). For details of puckering analysis, see Cremer & Pople (1975 ▶).
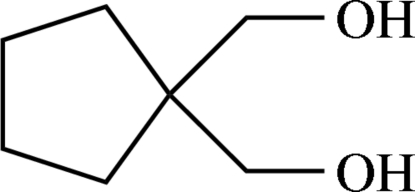


## Experimental

### 

#### Crystal data

C_7_H_14_O_2_
*M* *_r_* = 130.18
Monoclinic, 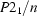

*a* = 5.8614 (16) Å
*b* = 10.631 (3) Å
*c* = 11.917 (3) Å
β = 98.33 (2)°
*V* = 734.7 (3) Å^3^
*Z* = 4
Mo *K*α radiation
μ = 0.08 mm^−1^
*T* = 200 (2) K
0.20 × 0.17 × 0.06 mm

#### Data collection

Oxford Diffraction Xcalibur diffractometer
Absorption correction: none
4224 measured reflections
1692 independent reflections
924 reflections with *I* > 2σ(*I*)
*R*_int_ = 0.067

#### Refinement

*R*[*F* ^2^ > 2σ(*F* ^2^)] = 0.061
*wR*(*F* ^2^) = 0.149
*S* = 1.01
1692 reflections
85 parameters
H-atom parameters constrained
Δρ_max_ = 0.19 e Å^−3^
Δρ_min_ = −0.17 e Å^−3^

### 

Data collection: *CrysAlis CCD* (Oxford Diffraction, 2005 ▶); cell refinement: *CrysAlis RED* (Oxford Diffraction, 2005 ▶); data reduction: *CrysAlis RED*; program(s) used to solve structure: *SHELXS97* (Sheldrick, 2008 ▶); program(s) used to refine structure: *SHELXL97* (Sheldrick, 2008 ▶); molecular graphics: *ORTEP-3* (Farrugia, 1997 ▶); software used to prepare material for publication: *SHELXL97*.

## Supplementary Material

Crystal structure: contains datablocks global, I. DOI: 10.1107/S1600536809001330/zl2170sup1.cif

Structure factors: contains datablocks I. DOI: 10.1107/S1600536809001330/zl2170Isup2.hkl

Additional supplementary materials:  crystallographic information; 3D view; checkCIF report

## Figures and Tables

**Table 1:** Hydrogen-bond geometry (Å, °)

*D*—H⋯*A*	*D*—H	H⋯*A*	*D*⋯*A*	*D*—H⋯*A*
O1—H1⋯O2^i^	0.84	1.91	2.720 (2)	163
O2—H2⋯O1^ii^	0.84	1.88	2.691 (2)	161

Symmetry codes: (i) 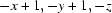; (ii) 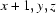.

**Fig. 1.**
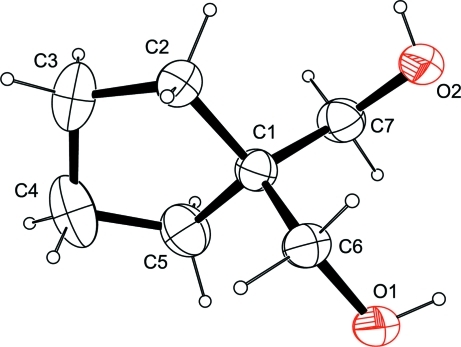
The molecular structure of the title compound, with atom labels and anisotropic displacement ellipsoids (drawn at 50% probability level) for non-H atoms.

**Fig. 2.**
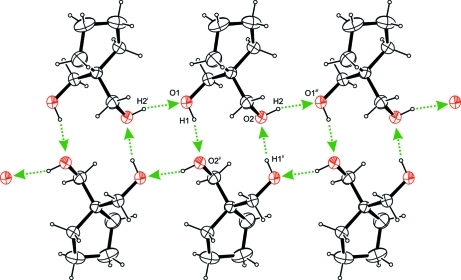
Hydrogen bonds in the crystal structure of the title compound, viewed along [0 1 0]. Symmetry operators: ix - 1, y, z; ii -x + 1, -y + 1, -z; iiix + 1, y, z.

**Fig. 3.**
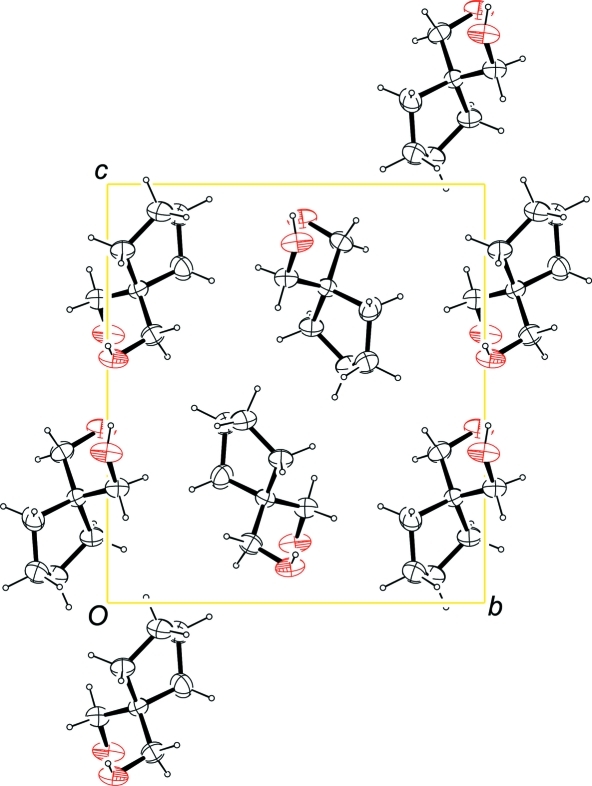
The packing of the title compound, viewed along [-1 0 0].

## supplementary crystallographic information

### Comment

In a program focused on the influence of chelation to (semi-)metals on the geometry of bifunctional alcohols (Klüfers & Vogler, 2007), the structure of 1,1-bis(hydroxymethyl)cyclopentane was elucidated.

Neglecting the hydrogen atoms of the hydroxy groups, the molecule would show non-crystallographic *C*_2_ symmetry (Fig. 1).

According to a conformational analysis (Cremer & Pople, 1975), the cyclopentane-moiety adopts an *envelope* conformation ^C4^*E* (*Q*_2_ = 0.404 (3) Å), which is slightly distorted towards a *twist* conformation ^C4^*T*_C3_ (*φ*_2_ = 280 (4)°).

In the crystals structure, hydrogen bonds furnish the formation of cooperative eight-membered homodromic rings (Fig. 2). These connect the molecules to strands along [1 0 0]. In terms of graph-set analysis (Etter *et al.*, 1990; Bernstein *et al.*, 1995), the descriptor for this pattern is *R*_4_^4^(8).

The molecular packing of the compound is shown in Figure 3.

### Experimental

The compound was prepared upon reacting 1,4-dibromobutane with malonic acid diethylester under basic conditions according to a published procedure (Domin *et al.*, 2005). Crystals suitable for X-ray analysis were obtained upon recrystallization of the crude reaction product from a boiling mixture of ethyl acetate - light petrol ether (1:1).

### Refinement

All H-atoms were placed in calculated positions (C—H 0.99 Å and O—H 0.84 Å) and were included in the refinement in the riding model approximation, with *U*(H) set to 1.2*U*_eq_(C) for methylene groups and *U*(H) set to 1.5*U*_eq_(O). Hydroxyl H atoms were allowed to rotate with a fixed angle around the C-O bond to best fit the experimental electron density (HFIX 147 in the *SHELX* program suite (Sheldrick, 2008)).

### Crystal data

**Table:**

C_7_H_14_O_2_	*Z* = 4
*M**_r_* = 130.18	*F*(000) = 288
Monoclinic, *P*2_1_/*n*	*D*_x_ = 1.177 Mg m^−^^3^
Hall symbol: -P 2yn	Mo *K*α radiation, λ = 0.71073 Å
*a* = 5.8614 (16) Å	θ = 4.6–27.5°
*b* = 10.631 (3) Å	µ = 0.08 mm^−^^1^
*c* = 11.917 (3) Å	*T* = 200 K
β = 98.33 (2)°	Platelet, colourless
*V* = 734.7 (3) Å^3^	0.20 × 0.17 × 0.06 mm

### Data collection

**Table:**

Oxford Diffraction Xcalibur diffractometer	924 reflections with *I* > 2σ(*I*)
Radiation source: fine-focus sealed tube	*R*_int_ = 0.067
graphite	θ_max_ = 27.5°, θ_min_ = 4.6°
ω scans	*h* = −7→4
4224 measured reflections	*k* = −13→13
1692 independent reflections	*l* = −14→15

### Refinement

**Table:**

Refinement on *F*^2^	Primary atom site location: structure-invariant direct methods
Least-squares matrix: full	Secondary atom site location: difference Fourier map
*R*[*F*^2^ > 2σ(*F*^2^)] = 0.061	Hydrogen site location: inferred from neighbouring sites
*wR*(*F*^2^) = 0.149	H-atom parameters constrained
*S* = 1.01	*w* = 1/[σ^2^(*F*_o_^2^) + (0.0527*P*)^2^] where *P* = (*F*_o_^2^ + 2*F*_c_^2^)/3
1692 reflections	(Δ/σ)_max_ < 0.001
85 parameters	Δρ_max_ = 0.19 e Å^−^^3^
0 restraints	Δρ_min_ = −0.17 e Å^−^^3^

### Fractional atomic coordinates and isotropic or equivalent isotropic displacement parameters (Å^2^)

**Table:**

	*x*	*y*	*z*	*U*_iso_*/*U*_eq_	
O1	0.2142 (2)	0.49721 (17)	0.14033 (13)	0.0473 (5)	
H1	0.2522	0.5090	0.0758	0.071*	
O2	0.7532 (3)	0.48406 (16)	0.08445 (12)	0.0431 (5)	
H2	0.8893	0.4984	0.1146	0.065*	
C1	0.5747 (4)	0.41846 (19)	0.24878 (17)	0.0299 (5)	
C2	0.7724 (4)	0.4594 (2)	0.34277 (18)	0.0381 (6)	
H21	0.9204	0.4638	0.3122	0.046*	
H22	0.7393	0.5432	0.3729	0.046*	
C3	0.7843 (5)	0.3602 (2)	0.4357 (2)	0.0562 (8)	
H31	0.8906	0.2912	0.4223	0.067*	
H32	0.8361	0.3975	0.5112	0.067*	
C4	0.5393 (6)	0.3131 (3)	0.4270 (2)	0.0586 (8)	
H41	0.4413	0.3733	0.4619	0.070*	
H42	0.5330	0.2302	0.4641	0.070*	
C5	0.4640 (4)	0.3034 (2)	0.2995 (2)	0.0475 (7)	
H51	0.2939	0.3064	0.2812	0.057*	
H52	0.5198	0.2240	0.2696	0.057*	
C6	0.4039 (4)	0.5258 (2)	0.22489 (19)	0.0363 (6)	
H61	0.3447	0.5481	0.2960	0.044*	
H62	0.4853	0.6002	0.2004	0.044*	
C7	0.6631 (4)	0.3799 (2)	0.14014 (19)	0.0388 (6)	
H71	0.7856	0.3158	0.1581	0.047*	
H72	0.5356	0.3410	0.0880	0.047*	

### Atomic displacement parameters (Å^2^)

**Table:**

	*U*^11^	*U*^22^	*U*^33^	*U*^12^	*U*^13^	*U*^23^
O1	0.0243 (8)	0.0853 (13)	0.0320 (8)	0.0014 (8)	0.0036 (7)	0.0073 (9)
O2	0.0276 (8)	0.0721 (12)	0.0303 (8)	−0.0023 (9)	0.0060 (7)	0.0062 (8)
C1	0.0290 (11)	0.0332 (11)	0.0274 (11)	−0.0025 (10)	0.0043 (9)	−0.0028 (9)
C2	0.0331 (12)	0.0496 (14)	0.0308 (11)	−0.0007 (11)	0.0018 (10)	−0.0010 (10)
C3	0.074 (2)	0.0586 (17)	0.0326 (13)	0.0067 (16)	−0.0032 (14)	0.0047 (12)
C4	0.093 (2)	0.0465 (15)	0.0408 (14)	−0.0035 (16)	0.0266 (15)	0.0052 (12)
C5	0.0526 (16)	0.0417 (14)	0.0500 (15)	−0.0048 (13)	0.0139 (13)	0.0066 (12)
C6	0.0288 (11)	0.0470 (14)	0.0330 (11)	0.0021 (11)	0.0039 (10)	0.0001 (10)
C7	0.0348 (12)	0.0454 (13)	0.0370 (13)	0.0008 (11)	0.0074 (11)	−0.0068 (11)

### Geometric parameters (Å, °)

**Table:**

O1—C6	1.421 (3)	C3—H31	0.9900
O1—H1	0.8400	C3—H32	0.9900
O2—C7	1.431 (3)	C4—C5	1.523 (4)
O2—H2	0.8400	C4—H41	0.9900
C1—C6	1.517 (3)	C4—H42	0.9900
C1—C7	1.519 (3)	C5—H51	0.9900
C1—C5	1.548 (3)	C5—H52	0.9900
C1—C2	1.553 (3)	C6—H61	0.9900
C2—C3	1.524 (3)	C6—H62	0.9900
C2—H21	0.9900	C7—H71	0.9900
C2—H22	0.9900	C7—H72	0.9900
C3—C4	1.510 (4)		
			
C6—O1—H1	109.5	C5—C4—H41	111.2
C7—O2—H2	109.5	C3—C4—H42	111.2
C6—C1—C7	109.90 (18)	C5—C4—H42	111.2
C6—C1—C5	111.40 (18)	H41—C4—H42	109.1
C7—C1—C5	109.48 (18)	C4—C5—C1	104.99 (19)
C6—C1—C2	109.13 (17)	C4—C5—H51	110.7
C7—C1—C2	112.31 (17)	C1—C5—H51	110.7
C5—C1—C2	104.54 (18)	C4—C5—H52	110.7
C3—C2—C1	106.30 (19)	C1—C5—H52	110.7
C3—C2—H21	110.5	H51—C5—H52	108.8
C1—C2—H21	110.5	O1—C6—C1	113.57 (18)
C3—C2—H22	110.5	O1—C6—H61	108.9
C1—C2—H22	110.5	C1—C6—H61	108.9
H21—C2—H22	108.7	O1—C6—H62	108.9
C4—C3—C2	103.7 (2)	C1—C6—H62	108.9
C4—C3—H31	111.0	H61—C6—H62	107.7
C2—C3—H31	111.0	O2—C7—C1	112.37 (18)
C4—C3—H32	111.0	O2—C7—H71	109.1
C2—C3—H32	111.0	C1—C7—H71	109.1
H31—C3—H32	109.0	O2—C7—H72	109.1
C3—C4—C5	103.1 (2)	C1—C7—H72	109.1
C3—C4—H41	111.2	H71—C7—H72	107.9
			
C6—C1—C2—C3	125.3 (2)	C2—C1—C5—C4	19.5 (2)
C7—C1—C2—C3	−112.5 (2)	C7—C1—C6—O1	57.0 (2)
C5—C1—C2—C3	6.1 (2)	C5—C1—C6—O1	−64.6 (2)
C1—C2—C3—C4	−29.5 (2)	C2—C1—C6—O1	−179.48 (17)
C2—C3—C4—C5	41.6 (3)	C6—C1—C7—O2	53.2 (2)
C3—C4—C5—C1	−38.0 (3)	C5—C1—C7—O2	175.88 (19)
C6—C1—C5—C4	−98.3 (2)	C2—C1—C7—O2	−68.5 (2)
C7—C1—C5—C4	140.0 (2)		

### Hydrogen-bond geometry (Å, °)

**Table:**

*D*—H···*A*	*D*—H	H···*A*	*D*···*A*	*D*—H···*A*
O1—H1···O2^i^	0.84	1.91	2.720 (2)	163
O2—H2···O1^ii^	0.84	1.88	2.691 (2)	161

Symmetry codes: (i) −*x*+1, −*y*+1, −*z*; (ii) *x*+1, *y*, *z*.
